# The impact of acute remote ischaemic preconditioning on cerebrovascular function

**DOI:** 10.1007/s00421-019-04297-1

**Published:** 2020-01-13

**Authors:** Howard H. Carter, Joseph D. Maxwell, Ylva Hellsten, Andrew Thompson, Dick H. J. Thijssen, Helen Jones

**Affiliations:** 1 Department of Nutrition, Exercise and Sports, Integrative Physiology Group, University of Copenhagen, Copenhagen, Denmark; 2 Research Institute of Sport and Exercise Science, Liverpool John Moores University, Tom Reilly Building, Byrom Street, Liverpool, L3 3AF UK; 3 Institute of Translational Medicine, University of Liverpool, Liverpool, UK; 4 Department of Physiology, Radboud Institute of Health Sciences, Radboud University Medical Centre, Nijmegen, The Netherlands

**Keywords:** Remote ischaemic preconditioning, Cerebral autoregulation, Cerebral blood flow, Hypercapnia

## Abstract

**Purpose:**

Remote ischaemic preconditioning (RIPC) refers to the protection conferred to tissues and organs via brief periods of ischaemia in a remote vascular territory, including the brain. Recent studies in humans report that RIPC provides neuroprotection against recurrent (ischaemic) stroke. To better understand the ability of RIPC to improve brain health, the present study explored the potential for RIPC to acutely improve cerebrovascular function.

**Methods:**

Eleven young healthy (females *n* = 6, age; 28.1 ± 3.7 years) and 9 older individuals (females *n* = 4, age 52.5 ± 6.7 years) at increased risk for stroke (cardiovascular disease risk factors) underwent assessments of cerebrovascular function, assessed by carbon dioxide (CO_2_) reactivity and cerebral autoregulation during normo- and hypercapnia (5% CO_2_) following 40 mins of bilateral arm RIPC or a sham condition. Squat-to-stand manoeuvres were performed to induce changes in blood pressure to assess cerebral autoregulation (0.10 Hz) and analysed via transfer function.

**Results:**

We found no change in middle cerebral artery velocity or blood pressure across 40 mins of RIPC. Application of RIPC resulted in no change in CO_2_ reactivity slopes (sham vs RIPC, 1.97 ± 0.88 vs 2.06 ± 0.69 cm/s/mmHg *P* = 0.61) or parameters of cerebral autoregulation during normocapnia (sham vs RIPC, normalised gain%, 1.27 ± 0.25 vs 1.22 ± 0.35, *P* = 0.46).

**Conclusion:**

This study demonstrates that a single bout of RIPC does not influence cerebrovascular function acutely in healthy individuals, or those at increased cardiovascular risk. Given the previously reported protective role of RIPC on stroke recurrence in humans, it is possible that repeated bouts of RIPC may be necessary to impart beneficial effects on cerebrovascular function.

## Introduction

Remote ischaemic preconditioning (RIPC) is a technique that offers enhanced hypoxic tolerance and protection to systemic organs and tissues following repeated brief periods of ischaemia and reperfusion to a remote vascular bed (Lim and Hausenloy 2012). This phenomenon, mediated via a neural and/or humoral pathway (Shimizu et al. 2009; Jensen et al. 2012), was first described in canine hearts (Przyklenk et al. 1993) with subsequent studies demonstrating its efficacy in humans. More specifically, RIPC has been reported to reduce cardiovascular events in patients following coronary artery bypass and percutaneous coronary intervention surgeries (Thielmann et al. 2013; Davies et al. 2013), and reduce brachial artery endothelial ischemia reperfusion damage (Kharbanda et al. 2002). Given these broad potent protective effects, it is possible that RIPC may also affect the brain and cerebral vasculature.

Animal studies have reported RIPC-mediated neuroprotection in the form of reduced infarct size and improved neurological recovery following prolonged cerebral ischaemia and hypothermic circulatory arrest (Jensen et al. 2011; Ren et al. 2008). Extending these findings to humans, a study in patients with aneurysmal subarachnoid haemorrhage reported 3 to 4 bouts of RIPC within 2–12 days post event induced changes indicative of cerebral vasodilation (via morphological clustering and analysis of intracranial pulse) (Gonzalez et al. 2013). A study in stroke survivors reported increased cerebral perfusion and 70% lower stroke recurrence following daily RIPC for 300 days, compared to a group of patients receiving standard care (Meng et al. 2012). This protective effect was reinforced in a recent study in acute stroke patients where repeated application of RIPC significantly improved clinical status and reduced National Institutes of Health Stroke Scale scores (England et al. 2017), while RIPC was found to significantly reduce white matter hyperintensities volume in small vessel disease patients (Wang et al. 2017). Strict regulation of brain blood flow in response to metabolic demand and stimuli such as blood pressure and arterial blood gases is crucial for the maintenance of cerebrovascular health and is impaired in numerous clinical groups, including stroke survivors. Based on previous observations that repeated RIPC improves peripheral macro- and microvascular health in humans (Kharbanda et al. 2002; Jones et al. 2014), the observed benefits of RIPC on cerebrovascular health may be related to acute improvements in cerebrovascular function in vivo. Assessing the impact of RIPC on cerebrovascular function would (1) extend our fundamental understanding of the acute effects of RIPC in humans, and (2) may provide insight into how RIPC mediates neuroprotection and further establish it as a novel therapeutic strategy in clinically vulnerable groups.

The primary aim of this proof of principle study was to assess the impact of bilateral arm RIPC on resting cerebral blood velocity and cerebral vascular function as assessed by cerebral autoregulation and cerebral vascular reactivity to carbon dioxide (CO_2_) in healthy individuals, compared to a sham condition. The CO_2_ reactivity assessment was selected based on previous studies suggesting it is an indicator of cerebral endothelial function (Lavi et al. 2006; Hoiland et al. 2017), while dynamic cerebral autoregulation is an indicator of cerebral vascular health and impaired in patients with cardiovascular disease when compared to healthy individuals (Caldas et al. 2016). To examine the effectiveness of RIPC across a broader spectrum of vascular health, we also included participants at an increased risk for cardiovascular disease (CVD) and stroke. Finally, previous studies have reported that hypercapnia (induced by inhalation of higher concentrations of CO_2_) transiently disrupts cerebral autoregulation and has been used as a model for impaired autoregulation (Jeong et al. 2016; Panerai et al. 1999; Zhang et al. 1998; Ainslie et al. 2008). Therefore, the secondary aim of this study was to assess the ability of RIPC to attenuate hypercapnia-induced impairment of cerebral autoregulation. We hypothesised that RIPC would improve cerebral autoregulation and CO_2_ reactivity, while attenuating the hypercapnia-induced impairment in cerebral autoregulation, when compared to a sham condition in both young healthy individuals and those with increased cardiovascular risk.

## Materials and methods

### Participants

Twenty participants were recruited for the study [healthy; *n* = 11 (females *n* = 6) and CVD risk; *n* = 9 (females *n* = 4), Table 1]. Healthy young participants (age 28 ± 4 years) were recreationally active, engaged in low-to-moderate intensity exercise 2–3 days per week, and were free from cardiovascular diseases, including diabetes, hypertension or hypercholesterolemia. For the second group, older individuals (53 ± 7 years) with cardiovascular risk factors were recruited based on having ≥ 1 of the following criteria; body mass index > 30 g/m^2^ or a waist circumference ≥ 94 cm (male), ≥ 80 cm (female), blood pressure systolic > 130/diastolic > 85 mmHg or diagnosed with high cholesterol (total > 200 mg/dL, triglycerides > 150 mg/dL, LDL > 100 mg/dL). Smokers, individuals with angina, heart failure or a history of myocardial infarction, transient ischaemic attack or stroke and thrombosis were excluded from participation. Participants were informed of the study protocol verbally and in writing before providing written informed consent. The study was approved by the University Research Ethics Committee and adhered to the standards set out in the Declaration of Helsinki.

**Table 1:** Group characteristics

Characteristics	Healthy individuals, *n* = 11; female = 6	CVD risk individuals, *n* = 9; female = 4	*P* value
Age (years)	28 ± 4	53 ± 7	< 0.001
Height (cm)	173.1 ± 10.1	169.4 ± 10.3	0.44
Weight (kg)	71.7 ± 13.6	93.6 ± 23.9	0.02
BMI (kg/m^2^)	24 ± 3	32 ± 6	< 0.001
MAP (mmHg)	89 ± 4	104 ± 3	< 0.001
PetCO_2_ (mmHg)	37.8 ± 2.0	40.2 ± 2.9	0.10
MCAv (cm s^−1^)	70 ± 15	54 ± 8	0.02

Values are means ± SD

*BMI* body mass index, *MAP* mean arterial pressure, *PetCO*_*2*_ partial pressure of end tidal carbon dioxide, *MCAv* middle cerebral artery velocity

### Study design

**Fig. 1:**
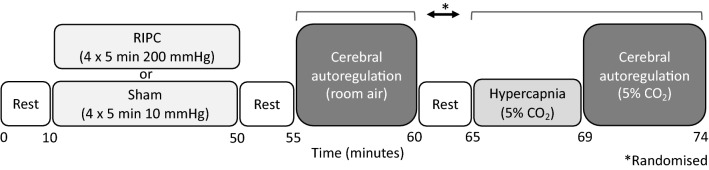
Schematic of the protocol for each testing visit

Participants attended the laboratory on two occasions (separated by a minimum of 3 days). All tests were performed at the same time of day to control for diurnal variation in cerebrovascular function (Ainslie et al. 2007). All participants arrived at the laboratory following an overnight fast and had refrained from alcohol, exercise and caffeine for 24 h prior to each visit. Visits were randomised and counterbalanced to receive either the bilateral upper arm RIPC or the sham condition. Each visit consisted of the bilateral assessment of middle cerebral blood velocity (MCAv) during RIPC or sham. Following this cerebral autoregulation was assessed using a 5 min squat-stand protocol (0.10 Hz). This was then proceeded by a 5 min rest period, followed by 4 min of hypercapnia (5% CO_2_) and then another 5 min squat-stand (0.10 Hz) protocol but whilst breathing 5% CO_2_ (See Fig. 1). The phase of menstrual cycle was not controlled for in the female participants.

### Study procedures

#### Remote ischaemic preconditioning and sham

The RIPC condition consisted of 8 bouts in total involving the inflation of a pneumatic cuff (Hokanson SC10D; USA) on the upper arm using a rapid inflator (EC-20; D.E Hokanson) to 220 mmHg for 5 min. This protocol was based on a previous study that revealed 4 × 5 min bouts of occlusion and reperfusion on alternate limbs induced a greater improvement in exercise performance compared to unilateral occlusion and reperfusion (Cocking et al. 2018). Cuffs were inflated in an alternating fashion allowing for one arm to be occluded while the contralateral arm underwent reperfusion. The sham condition consisted of the identical protocol with the difference that the cuff pressure was inflated to only 10 mmHg.

#### Cerebral blood flow (middle cerebral artery blood velocity)

Following 20 min rest in the supine position, bilateral MCAv’s were continuously measured through the temporal window using transcranial Doppler ultrasonography (TCD). Two 2-MHz Doppler probes (Spencer Technologies, Seattle, USA) were adjusted until an optimal signal was identified and held in place using a Marc 600 head frame (Spencer Technologies, Seattle, USA). Once the optimal MCAv signal was attained, the probe location and machine settings (depth, gain and power) were recorded to identify the same imaging site for the second testing session. Participants were instrumented with a two-way valve mouthpiece (Hans Rudolph) from which end tidal CO_2_ (P_ET_CO_2_) was measured using a calibrated gas analyser (ML206 ADinstruments, Colorado Springs, USA). Continuous beat-by-beat blood pressure was obtained from a digit (Finapres, Amsterdam, Netherlands) and heart rate acquired from a 3 lead electrocardiogram. All data was sampled at 50 Hz with the data acquisition system PowerLab via the interface LabChart 7 (ADinstruments, Colorado Springs, USA).

#### Cerebral autoregulation

Dynamic cerebral autoregulation was assessed using a squat-to-stand procedure that induces transient changes in arterial blood pressure (Claassen et al. 2009; Smirl et al. 2015). Participants replicated the experimenter whilst performing the manoeuvres that involved moving from a standing upright position to squatting until the legs achieved a 90° angle. Participants performed two sets at 0.10 Hz (5 s squat–5 s stand) while breathing normal atmospheric air, and again during hypercapnia (detailed below). The first set of squat-stands was preceded by 5 min of seated rest while the second set immediately followed the 4 min of hypercapnia.

#### Carbon dioxide reactivity

Following a rest period of 5 min, a baseline measurement of cerebral blood velocity, MAP and P_ET_CO_2_ was performed across 2 min while participants breathed in room air. Following the baseline period, the inhaled air was switched to a Douglas bag (100 L) containing 5% CO_2_, 21% oxygen and balanced nitrogen, while participants sat in a rested seated position.

### Data analysis

MCAv and MAP during the 40 min RIPC and sham conditions were averaged and extracted from LabChart in 5 min intervals (*n* = 20). MCA cerebrovascular conductance (CVC) was calculated as MCAv/mean arterial pressure (MAP). Calculation of the cerebrovascular CO_2_ reactivity slopes were performed via linear regression analysis of the two time-points; baseline (MCAv, MAP, P_ET_CO_2_ averaged across 2 min) and 5% CO_2_ (data averaged across the last 30 s of the 4 min hypercapnia). Two participants in the cardiovascular risk factor group were unable to complete the hypercapnic protocol, therefore data analysis for CO_2_ reactivity was performed on *n* = 18 (Healthy = 11).

Cerebral autoregulation data were extracted from LabChart beat-to-beat (MAP and MCAv) before spline interpolation and assessed via transfer function analysis (TFA) based on the Welch algorithm, using a provided script (https://www.car-net.org/). The 5 min squat-stand recordings were subdivided into five windows overlapping by 50% and passed through a Hanning window before fast Fourier transform analysis (MathWorks-Inc., Natick, Massachusetts). The cross-spectrum between MAP and MCAv was determined and divided by MAP auto-spectrum to formulate functions; normalised gain, absolute gain, phase and coherence (MAP-MCAv linearity). Gain represents the difference in amplitudes between the cerebral blood velocity and blood pressure signals, while phase describes the temporal alignment between the input (MAP) and output (MCAv). Gain and phase data were excluded from statistical analysis if coherence was < 0.4. TFA was performed in accordance with standardised guidelines from the Cerebral Autoregulation Research Network (Claassen et al. 2016). TFA parameters of the driven oscillations were band averaged across the very low (VLF; 0.02–0.07 Hz), low (LF; 0.07–0.2 Hz) and high (HF; 0.2–0.4 Hz) frequency domains. We induced BP oscillations at 0.10 Hz in the current study, this falls within the ranges of the LF domain. Therefore, the low frequency (0.07–0.20 Hz) output is the most appropriate to be reported as cerebral autoregulation is highly active with this frequency of squats (Zhang et al. 1998). P_ET_CO_2_ data was averaged across each 5 min squat-stand recording. One participant in the cardiovascular risk factor group was unable to complete the cerebral autoregulation protocol during normocapnia while three participants from the same group were unable to complete the protocol during hypercapnia, therefore data was analysed on *n* = 18 for the normocapnic and *n* = 17 for the hypercapnic cerebral autoregulation conditions.

### Statistical analysis

A three-factor group × condition × time (group; healthy vs CVD risk factors, condition: RIPC vs sham, time: 5 min intervals during intervention) general linear model was employed to analyse resting MCAv and MAP during the RIPC and sham intervention. A three-factor -capnia × group × condition (capnia; normocapnic or hypercapnic, group; healthy vs CVD risk factors, condition: RIPC vs sham) general linear model was employed to analyse the cerebral autoregulation. Hypercapnic CO_2_ reactivity responses were analysed via a linear mixed model and assessed for a three-way interaction (group × condition × PetCO_2_). MCAv was entered as the outcome variable, with PetCO_2_ as a predictor variable and MAP as a covariate. PetCO_2_ was also entered as a random factor in the model. Statistically significant main effects and interactions were followed up with the least significant difference (LSD) approach for multiple comparisons. Statistical analysis was conducted using Statistical Package for Social Sciences (Version 22; SPSS Inc., Chicago, IL, USA). Statistical significance was delimited at *P* < 0.05. Data are presented in the text as mean (95% confidence interval) unless otherwise stated.

## Results

### Group characteristics

Resting MCAv was significantly higher in the healthy compared to CVD risk group (Table 1, *P* = 0.02), while resting MAP was significantly lower in the healthy compared to the CVD risk group (Table 1, *P* = 0.001). For resting comparisons of cerebrovascular function between the groups, responses to the CO_2_ reactivity and cerebral autoregulation tests during the sham condition are reported. No difference was evident in CO_2_ reactivity slopes between the healthy and CVD risk groups at rest [2.15 (1.60, 2.70) vs 1.68 (1.13, 2.24) cm/s/mmHg, *P* = 0.44], or for any of the dynamic cerebral autoregulation variables (Table 2).

**Table 2:** Cerebral autoregulation analysis via transfer function using squat-stand manoeuvres

	Healthy	CVD risk	*P* value
Sham condition—normocapnia			
PetCO_2_ (mmHg)	37.46 ± 1.89	40.28 ± 3.19	0.10
Dynamic cerebral autoregulation (0.10 Hz)			
MCAv power (cm/s)^2^	88.78 ± 39.65	64.89 ± 80.16	0.16
MAP power (mmHg^2^)	118.91 ± 50.60	87.01 ± 80.16	0.12
Normalised gain %	1.25 ± 0.30	1.30 ± 0.20	0.87
Gain (cm/s/mmHg)	0.84 ± 0.19	0.74 ± 0.12	0.46
Phase (radians)	0.55 ± 0.37	0.60 ± 0.55	0.89
Coherence	0.61 ± 0.10	0.67 ± 0.09	0.12

Resting comparison of healthy and CVD risk participants

Values are means ± SD

### Impact of RIPC on resting cerebral blood velocity and haemodynamics

**Fig. 2:**
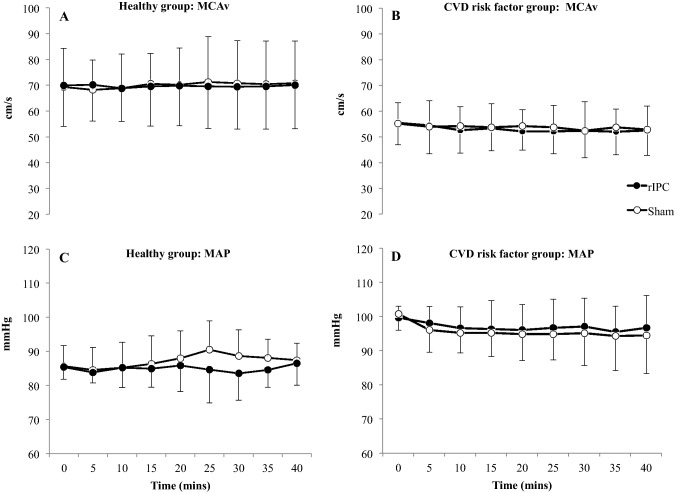
Middle cerebral artery velocity (MCAv) in young healthy (**a**), older cardiovascular risk factor (**b**) individuals and mean arterial pressure (MAP) in young healthy (**c**) and cardiovascular risk factor (**d**) individuals during 40 min of RIPC and sham. Data is mean ± SD

There was no impact of RIPC on MCAv across the 40 min (Fig. 2, *P* = 0.58). There was a group*condition interaction, with MAP being higher during RIPC compared to sham in the CVD risk group over the 40 min intervention period (P < 0.005), whilst MAP was similar between conditions in the healthy group.

### Impact of RIPC on cerebrovascular CO_2_ reactivity

The inhalation of 5% CO_2_ significantly increased PetCO_2_ following the sham and RIPC conditions, respectively (Table 3, both *P* < 0.001). MCAv subsequently increased, with no difference between the sham and RIPC conditions (Table 3, *P* = 0.43). There was no overall effect of RIPC on CO_2_ reactivity compared to the sham condition (group × treatment × PetCO_2_, *P* = 0.61, Table 3).

**Table 3:** Cardiovascular and respiratory parameters during the carbon dioxide reactivity test

Healthy + CVD Risk	Sham		RIPC		Sham v RIPC *P* value
	Baseline	5% CO_2_	Baseline	5% CO_2_	
MCAv (cm/s)	64 ± 12	83 ± 19*	63 ± 11	83 ± 18*	
PetCO_2_ (mmHg)	34 ± 6	44 ± 3*	34 ± 6	44 ± 3*	
MAP (mmHg)	101 ± 8	108 ± 9*	100 ± 5	106 ± 6*	
MCA reactivity to CO_2_ (cm/s/mmHg)	1.97 ± 0.88		2.06 ± 0.69		0.61

Values are means ± SD. Data are presented grouped (Healthy and CVD risk) as there were no significant interactions between participant groups. Statistical significance was set at *P* < 0.05

*MCAv* middle cerebral artery velocity, *PetCO*_*2*_ partial pressure of end tidal carbon dioxide, *MAP* mean arterial pressure

^*^Statistically significant from baseline at *P* < 0.001

### Impact of RIPC on normocapnic and hypercapnic cerebral autoregulation

During normocapnia, there were no main effects or interactions in the low frequency (0.10 Hz) for normalised gain (Table 4, *P* = 0.46), phase (*P* = 0.53) or coherence (*P* = 0.59) between the sham and RIPC conditions. PetCO_2_ values during the squat-stand procedure were not different between conditions (*P* = 0.81).

**Table 4:** Transfer function analysis of oscillations in mean arterial pressure and middle cerebral artery velocity using squat-stand manoeuvres

Healthy + CVD risk	Normocapnia (*n* = 19; healthy = 11)			Hypercapnia (*n* = 17; healthy = 11)		
	Sham	RIPC	*P* value	Sham	RIPC	*P* value
PetCO_2_ (mmHg)	38.35 ± 2.65	38.15 ± 2.85	0.81	46.63 ± 2.74	46.46 ± 3.00	0.90
Dynamic cerebral autoregulation (0.10 Hz)						
MCAv power (cm/s)^2^	81.24 ± 54.43	80.95 ± 60.47	0.87	76.85 ± 40.04	82.82 ± 52.66	0.69
MAP power (mmHg^2^)	108.83 ± 55.61	117.67 ± 72.05	0.73	110.81 ± 62.25	119.47 ± 88.78	0.74
Normalised gain %	1.27 ± 0.25	1.22 ± 0.35	0.46	0.86 ± 0.16	0.94 ± 0.21	0.11
Gain (cm/s/mmHg)	0.80 ± 0.17	0.75 ± 0.17	0.86	0.75 ± 0.16	0.80 ± 0.20	0.82
Phase (radians)	0.53 ± 0.47	0.64 ± 0.39	0.53	0.38 ± 0.44	0.40 ± 0.31	0.90
Coherence	0.64 ± 0.10	0.65 ± 0.10	0.59	0.60 ± 0.20	0.58 ± 0.11	0.45

Comparison between Sham and RIPC conditions with all participants grouped together

Values are means ± SD. Data are presented grouped (healthy and CVD risk) as there were no significant interactions between participant groups. Statistical significance was set at *P* < 0.05

*PetCO*_*2*_ partial pressure of end tidal carbon dioxide, *MCAv* middle cerebral artery velocity, *MAP*, mean arterial pressure

Similarly, during hypercapnia, no significant main effects or interactions in the low frequency domains for normalised gain (Table 4, *P* = 0.11), phase (*P* = 0.90) or coherence (*P* = 0.45) were observed. PetCO_2_ values during hypercapnia did not differ between conditions (*P* = 0.90).

### Effect of hypercapnia on cerebral autoregulation (comparison of sham conditions)

Hypercapnia induced a phase reduction of 0.15 radians (0.08, 0.34) when compared to normocapnic cerebral autoregulation (*P* = 0.002). Additionally, normalised gain decreased during hypercapnic cerebral autoregulation by 0.41% (0.21, 0.47) compared to normocapnic (*P* < 0.001).

## Discussion

This is the first study to investigate the acute impact of RIPC on both dynamic cerebral autoregulation and cerebrovascular CO_2_ reactivity in healthy humans and those at increased risk of cardiovascular disease and stroke. Our principle findings are (1) resting cerebral blood velocity was significantly higher at baseline in the healthy group compared to the cardiovascular risk group and (2) RIPC did not impact resting cerebral perfusion, cerebrovascular CO_2_ reactivity or cerebral autoregulation, in either group. These findings extend our fundamental understanding of the acute effects of RIPC in humans and reveal that a single episode of RIPC does not immediately impact cerebrovascular function in humans.

Despite the well-documented effects of RIPC on myocardial and peripheral vascular function in humans (Bøtker et al. 2010; Thielmann et al. 2013; Davies et al. 2013; Kharbanda et al. 2002; Loukogeorgakis et al. 2005; Jones et al. 2014), the present study is the first to examine the acute impact of RIPC on cerebral blood velocity, and both cerebral autoregulation and CO_2_ reactivity in humans. The cerebral tests above were employed to provoke cerebral vasomotion via a number of different regulatory pathways, to better identify any specific effect RIPC may have. In response to 40 min of upper arm RIPC (4 bouts per arm, alternated), we observed no concurrent impact on cerebral blood velocity. Increases in arterial diameter and blood flow to limbs and organs (heart) regional to the limb undergoing RIPC have been previously reported during the reperfusion phases of RIPC (Enko et al. 2011; Zhou et al. 2007). Our finding that RIPC did not alter cerebral blood velocity during the bout is an important observation in this context, and suggests that RIPC does not influence blood vessel function similarly in the brain. Although it is not known what mechanism/s are responsible for the regional changes in blood flow in the previous studies during RIPC, we cannot discount the possibility that RIPC did induce a change in cerebral perfusion, and that this change was counteracted by one of the numerous cerebral blood flow regulatory mechanisms (Willie et al. 2014). However, consistent with the above finding of no change in blood flow, we observed no overall impact on cerebrovascular function. Resting cerebral autoregulation, a regulatory mechanism that maintains a constant delivery of oxygenated blood to the brain despite changes in blood pressure (Aaslid et al. 1989), was unchanged by RIPC. The second aim of this study was to temporarily disturb cerebral autoregulation via hypercapnia to determine whether RIPC could attenuate the impairment. As expected (Birch et al. 1995; Zhang et al. 1998; Panerai et al. 1999; Ainslie et al. 2008), hypercapnia reduced cerebral autoregulation phase (indicating a delayed CA response time), but did not alter absolute gain, an effect consistent with some (Ainslie et al. 2008), but not all studies (Jeong et al. 2016; Zhang et al. 1998; Panerai et al. 1999). However, in contrast to our hypothesis, RIPC did not attenuate the hypercapnia-induced impairment in phase (temporal alignment). Finally, RIPC did not impact cerebrovascular reactivity to inhalation of 5% CO_2_ compared to the sham condition. To our knowledge, there is only one directly relevant study that assessed RIPC and cerebrovascular function in humans (Rieger et al. 2017). In this study the authors measured cerebral blood flow responses to acute and chronic hypoxia, and found no effect of RIPC compared to controls, findings consistent with the present study. Despite this, there is increasing evidence that repeated RIPC is neuroprotective, particularly in clinical stroke and small vessels disease patients (Meng et al. 2012; England et al. 2017; Wang et al. 2017). Meng et al. previously reported that 300 days of repeated RIPC decreased stroke recurrence and interestingly noted that cerebral perfusion was higher in the RIPC group compared to the standard care patients, potentially remedying the mismatch between perfusion and metabolism. This study raises the intriguing notion that repeated bouts of RIPC may be required to influence cerebral perfusion and function to a physiologically relevant extent. Additionally, the phenomenon of RIPC-mediated protection is known to be biphasic in nature, with an immediate protective period that subsides within a few hours of application, followed by a more prolonged second protective window (1–3 days) (Koch et al. 2014). Due to the difficulties in assessing the time-course of RIPC effectiveness in humans, the vast majority of these studies have been performed in animals. Although we find this unlikely, one possible explanation for our null findings is that the cerebral measures were not performed within the initial protective phase, and that the protective windows in humans may differ to that of animals, and may also be influenced by the type, number and duration of RIPC bouts.

An important aspect to this study was assessing the impact of RIPC across a spectrum of cardiovascular health, to determine if this influenced the efficacy of RIPC. Young healthy individuals typically present with unimpaired endothelial-vascular function and as the magnitude of the RIPC effect on cerebrovascular function, if any, is unknown, it is possible that a RIPC effect would be not be observable in this population. Accordingly, we assessed the effect of RIPC in healthy individuals and those at increased cardio- and cerebrovascular risk. As expected, cardiovascular risk metrics were significantly different between the groups, with the young healthy individuals displaying lower mean arterial pressure and higher resting cerebral blood flows compared to the elevated risk individuals. Nonetheless, we observed no differences in the efficacy of RIPC to improve cerebral autoregulation under normo- and hypercapnic conditions between the groups.

We acknowledge the present study is not without limitations. Middle cerebral artery blood velocity was measured using transcranial Doppler, a technique that provides a reliable surrogate for absolute cerebral blood flow providing the insonated artery diameter remains constant across and between the study conditions (Ainslie and Hoiland 2014). Although unlikely, we cannot discount the possibility that RIPC induced a change in middle cerebral artery diameter that impacted our measures of cerebral blood flow. Our study included a mix of males and females which may have increased variability in our cerebral responses related to sex hormones (Krause et al. 2006). Additionally, the phase of the menstrual cycle in the female participants was not controlled, however as recent studies have reported that cerebral autoregulation and cerebral vascular reactivity to carbon dioxide remains unchanged across the menstrual cycle (Favre and Serrador 2019; Peltonen et al. 2016), it is unlikely it influenced our findings. Participants were screened for overt cardiovascular risk, however were not invasively screened for the presence of proximal cerebral stenosis or carotid artery disease, which if present may have impaired the cerebral autoregulatory responses. Finally, we recruited young healthy and older participants with CVD risk factors, meaning our results cannot be generalised to clinical populations. It is possible that RIPC may have had an observable effect in participants with clinical manifestation of cardio- or cerebrovascular disease and future studies will be required to determine this.

## Conclusion

The findings of this study extend our fundamental knowledge on the physiological effects of RIPC in humans by assessing for the first time the acute impact of RIPC on cerebral perfusion, cerebral autoregulation and CO_2_ reactivity. Although acute RIPC has been found to increase peripheral blood flow (limbs, heart), we report that this effect of RIPC does not extend to the cerebral circulation, as no change was observed in cerebral perfusion during RIPC. Additionally, RIPC did not influence cerebral function, as measured by autoregulation and cerebrovascular CO_2_ reactivity. With recent clinical trials showing that repeated RIPC provides neuroprotection in humans, future studies are required to determine whether repeated exposure to the RIPC stimulus is necessary to induce changes in cerebral function.

## Abbreviations

CO_2_: Carbon dioxide
CVC: Cerebrovascular conductance
CVD: Cardiovascular disease
LSD: Least significant difference
MAP: Mean arterial pressure
MCAv: Middle cerebral artery velocity
PetCO_2_: Partial pressure of carbon dioxide
TCD: Transcranial Doppler
TFA: Transfer function analysis
RIPC: Remote ischaemic preconditioning
